# Polyelectrolyte Gels: A Unique Class of Soft Materials

**DOI:** 10.3390/gels7030102

**Published:** 2021-07-24

**Authors:** Ferenc Horkay

**Affiliations:** Section on Quantitative Imaging and Tissue Sciences, Eunice Kennedy Shriver National Institute of Child Health and Human Development, National Institutes of Health, Bethesda, MD 20892, USA; horkayf@mail.nih.gov

**Keywords:** polyelectrolyte, gel, swelling, ions, volume phase transition, osmotic swelling pressure, elastic modulus, swelling kinetics

## Abstract

The objective of this article is to introduce the readers to the field of polyelectrolyte gels. These materials are common in living systems and have great importance in many biomedical and industrial applications. In the first part of this paper, we briefly review some characteristic properties of polymer gels with an emphasis on the unique features of this type of soft material. Unsolved problems and possible future research directions are highlighted. In the second part, we focus on the typical behavior of polyelectrolyte gels. Many biological materials (e.g., tissues) are charged (mainly anionic) polyelectrolyte gels. Examples are shown to illustrate the effect of counter-ions on the osmotic swelling behavior and the kinetics of the swelling of model polyelectrolyte gels. These systems exhibit a volume transition as the concentration of higher valence counter-ions is gradually increased in the equilibrium bath. A hierarchy is established in the interaction strength between the cations and charged polymer molecules according to the chemical group to which the ions belong. The swelling kinetics of sodium polyacrylate hydrogels is investigated in NaCl solutions and in solutions containing both NaCl and CaCl_2_. In the presence of higher valence counter-ions, the swelling/shrinking behavior of these gels is governed by the diffusion of free ions in the swollen network, the ion exchange process and the coexistence of swollen and collapsed states.

## 1. Introduction

It is often claimed that the gel state of a material is easier to recognize than to define. A widely accepted phenomenological definition is that a gel is a soft, solid or solid-like material of two or more components, one of which is a liquid, present in a substantial quantity [1,2]. From a rheological point of view, gels are characterized by the storage modulus, G’, which exhibits a plateau extending to times of the order of seconds, and by a loss modulus, G”, which is much smaller than the storage modulus in the plateau region. This definition is consistent with that of Ferry [3]. The term gel has also been used for systems that do not contain a liquid (e.g., vulcanized rubber or dried silica gel). 

Based on the mechanism of the cross-linking process, gels can be classified as physical or chemical networks [4,5]. Physical cross-links may arise from hydrogen bonding, hydrophobic interactions, inter-chain entanglements and crystallite formation. Physical cross-linking produces reversible gels. Although physical cross-links are not permanent, they are sufficiently strong to tie the polymer chains together. 

Chemically cross-linked gels can be formed by various chemical processes such as free radical polymerization, electromagnetic radiation (light, gamma, X-ray or electron beam) and chain or step-growth polymerization. In all cases, covalent cross-links alter the chemical structure of the polymer and have significant consequences on the physical properties of the system at both molecular and supramolecular levels. Cross-linking renders the polymer insoluble, and the type and extent of cross-linking influence important network properties, such as swelling, elastic and transport properties. In a given solvent, the degree of swelling depends on the cross-link density of the network and the interaction between the polymer and solvent. Uncross-linked polymers can be diluted infinitely. Cross-links prevent infinite swelling because the osmotic mixing pressure II_mix_, which is the driving force of swelling, is counter-balanced by the elastic pressure II_el_ generated by the cross-links. At equilibrium, the swollen network coexists with the pure solvent. 

Typical examples of physical gels are those formed by the cooling of solutions of biological or synthetic polymers (e.g., gelatin, agarose, polyvinyl alcohol). Typical chemical gels are dextrane gels (e.g., Sephadex), polyvinylalcohol gels, polystryrene gels, etc. Examples of biological gels include cartilage or fibrin clots formed by polymerization of fibrinogen monomers through enzymatic reactions. Silica gel is a typical example of an inorganic gel. The common feature of these materials is that they are soft, solid or solid-like and contain a liquid. Hydrogels are networks of hydrophilic polymers swollen in water. In organogels, the polymer network is hydrophobic, and the liquid is an organic solvent (e.g., toluene).

## 2. Effect of the Environment on the Swelling of Gels

The properties (e.g., swelling degree, elastic modulus) of many hydrogels are sensitive to changes in the environmental conditions (e.g., pH, ionic strength, solvent composition, temperature). For example, in pH- and ion-responsive gels, a volume transition can be induced by changing the ionization of the polyelectrolyte chains. By decreasing the degree of ionization, the electrostatic repulsion between the charged groups on the polymer chains is reduced, which ultimately leads to the collapse of the swollen network. In temperature-sensitive systems, the strength of polymer–solvent contacts varies relative to the polymer–polymer contacts, and the gel undergoes a volume transition. Light and the electric field can also induce changes in the swelling degree of gels. In the former, the volume change is due to the temperature increase caused by photosensitive groups (chromophores) which absorb light and dissipate heat. In electrosensitive gels, the applied electric field attracts mobile ions to the electrodes, and the hydrogel swells (or shrinks) at the cathode and the anode.

Temperature-sensitive hydrogels contain both hydrophilic and hydrophobic monomers. At low temperature, hydrogen bonding between the polymer and water molecules leads to dissolution in water. However, when the temperature exceeds a critical temperature (lower critical temperature, LCST), the hydrogen bonds break down, and phase separation occurs. Varying the relative amounts of hydrophilic and hydrophobic monomers can alter the LCST of hydrogels. 

Stimuli-responsive hydrogels are widely used in drug delivery devices to deliver drugs to a specific site in the body. These systems are frequently called smart or intelligent gels because the fast response to external stimuli is a typical feature of living systems [6,7,8,9]. Poly(N-isopropylacrylamide) (PNIPAM) is the most studied thermosensitive hydrogel in drug delivery applications. This is due to the ability of PNIPAM to squeeze out the absorbed drug when the temperature is near that of the human body. Glucose sensors are used in insulin delivery systems [10]. Another important application of smart gels is scaffolds for tissue engineering because they are capable of releasing cells in response to a stimulus [11,12]. 

## 3. Modeling of Polymer Gels

Modeling the response of gels to changes in the environmental conditions is a complex task that can be attempted at different length and time scales. The classical theory of Flory and Huggins describes gels in terms of osmotic and elastic forces, which define the degree of swelling [4]. High polymer–solvent affinity, i.e., a strong interaction among the polymer and solvent molecules, leads to a large osmotic pressure. The elastic contribution can be estimated from the theory of rubber elasticity [4,13]. In the case of charged polymer networks, electrostatic interactions and counter-ion osmotic pressure may also play a significant role [4].

Computer simulations provide valuable information on the behavior of polymer gels. There are various simulation methods to study polymer gels (e.g., Monte Carlo simulation, molecular dynamics, Brownian dynamics) [14,15,16,17,18,19]. The choice of the method depends on the focus of the study. The first step before conducting the simulation is to define the level of complexity required to capture the features of the system. It is common to use coarse graining to model polymer chains. The next step is to introduce intermolecular and intramolecular interactions and generate the initial configuration. Then, this configuration is relaxed, and the properties of the system are monitored until an equilibrium is attained. A typical approach is to study the dynamics of only a limited number of particles, while other particles (e.g., solvent molecules) are viewed as a continuous phase. This implicit solvent approach has the advantage of being computationally less expensive, thus making it possible to simulate larger systems over longer time scales. 

Over the past decade, computer simulation has proved to be a uniquely powerful tool in investigating polymer gels. It allows constructing near-perfect model networks with a well-defined topology. The network topology can be systematically varied from ideal to more realistic systems by introducing a controlled quantity of structural defects (entanglements, loops, dangling ends, etc.). In real gels, imperfections formed during the cross-linking process are always present. Computer simulation makes it possible to determine the effect of inhomogeneities on the material properties (e.g., mechanical strength). Earlier simulation studies largely focused on the dynamic properties of these systems, e.g., kinetics of cross-linking. Much less attempt has been made to simulate the swelling of gels that may require a very long time to reach an equilibrium. Future studies should focus, among others, on the effect of the size and structure of solvent molecules, the influence of the temperature on the interaction potentials and other molecular details of the network structure, such as network functionality and structural irregularities. 

## 4. Polyelectrolyte Gels

Polyelectrolytes are macromolecules containing ionizable groups, which, in aqueous solutions, dissociate and release counter-ions into the solution. These charged macromolecules play important roles in various processes in living systems such as DNA condensation, nerve excitation and load bearing of cartilage. Typical examples of biological polyelectrolytes are nucleic acids, proteins and proteoglycans. 

In general, in polyelectrolyte systems, electrostatic interactions between the polymer molecules and ions lead to a very rich behavior, which differs in many aspects from the behavior of neutral polymers. Polyelectrolyte gels contain charged groups on the cross-linked polymer chains, and ions in the swelling liquid [20,21,22]. They exhibit unique properties because the effects of ions on polyelectrolyte molecules are not only short range but, due to the connected structure of the polymer chains, also long range. Due to the presence of ionized groups and mobile ions, polyelectrolyte gels are sensitive to external stimuli such as changes in pH, ionic interactions and temperature, and they may exhibit a volume transition in response to these changes [23,24,25,26,27].

**Scheme 1:**
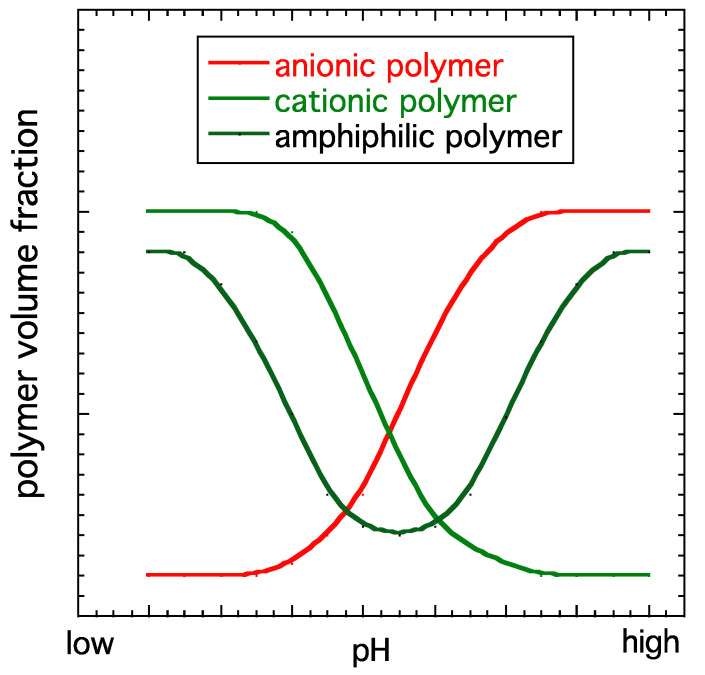
Schematic representation of the variation in the polymer volume fraction as a function of the pH in different types of polyelectrolyte gels. Anionic gels swell in basic solutions (high pH), and cationic gels swell in acidic solutions (low pH). Amphiphilic gels swell in both low- and high-pH solutions.

Polyelectrolyte gels have a large number of ionizable groups. They respond to the change in the pH in the surrounding liquid by either gaining or losing protons. In basic environments, anionic polyelectrolytes are deprotonated, and the strong electrostatic repulsion among the anionic chains leads to gel swelling. In acidic environments, the anionic polymer is protonated, resulting in a decrease in the charge density, and the gel collapses. Cationic polyelectrolytes exhibit the opposite behavior; they are ionized and swell in acidic environments and collapse in basic environments. There are polymers which contain both anionic and cationic groups. Such amphiphilic hydrogels swell in both acidic and basic environments. The pH dependence of the behavior of different charged hydrogels is illustrated in Scheme 1. 

Several theoretical models have been proposed to describe volume transitions in polyelectrolyte gels [28]. Katchalsky [29] extended the Flory–Rehner model developed for neutral polymer gels to describe the swelling of polyelectrolyte gels. It was shown that (i) the swelling of polyelectrolyte gels was governed by the balance between the elastic free energy of the cross-linked polymer chains and the osmotic pressure of the charged polymer and counter-ions in the gel, and (ii) a small variation in the salt concentration in the equilibrium solution could induce a volume transition in the polyelectrolyte gel. The condition of electroneutrality insured that the ion concentrations in the gel satisfied the Donnan equilibrium condition. 

The discontinuous volume transition in gels was first described by Dusek and Patterson [30] based on the analogy of the coil–globule transition of polymer chains in solution. The high swelling ability of polyelectrolyte gels reflects the electrostatic repulsion between the charged groups on the polymer chains. The addition of high-valence counter-ions screens the electrostatic interaction and leads to phase separation. Monovalent counter-ions are gradually replaced by high-valence counter-ions, and the osmotic pressure is reduced. At a critical threshold concentration of the multivalent ions, the osmotic pressure vanishes, and phase separation takes place.

Less attention has been paid to the kinetics of polyelectrolyte gel swelling. Gel swelling/shrinking involves the motion of both polymer and solvent molecules. The response time to changes in the environment (ion concentration and composition, temperature, pH, etc.) strongly depends on the actual geometry of the gel. Swelling kinetics measurements provide quantitative information on the collective diffusion coefficient. 

## 5. Gels in Living Systems

Biopolymers are naturally occurring macromolecules which are essential components of all living systems [31,32]. Many biopolymers are polyelectrolytes, i.e., charged macromolecules. Understanding the behavior of polyelectrolytes is one of the most challenging problems in polymer science [33,34,35]. The properties of these molecules reflect chain connectivity, electrostatic effects and various molecular interactions over multiple length scales. Owing to the complexity of these interactions, the progress in this field has been slow despite the importance of polyelectrolytes both in biology and materials science. 

In general, biological tissues are highly swollen polyelectrolyte gels. Many important tissue properties originate from the polyelectrolyte nature of their constituents. For example, articular cartilage consists of an extracellular matrix (ECM) containing negatively charged aggrecan/hyaluronic acid complexes embedded in a collagen matrix. In other biological systems, e.g., in the nervous system, Na^+^, K^+^ and Ca^2+^ ions regulate the excitability of neurons. Intracellular Ca^2+^ ions play an important role in a variety of physiological processes such as muscle contraction, hormone secretion, synaptic transmission and gene expression. 

Despite the many recent efforts in the field of polyelectrolytes, no satisfactory theoretical model exists that describes the behavior of these systems and provides quantitative agreement with the experimental findings. Problems such as counter-ion condensation, coupling between small ions and macro-ions and the effect of counter-ions on chain stiffness, which are necessary to understand the behavior of polyelectrolyte gels, have not yet been fully resolved. Polyelectrolyte gels have a great potential not only for designing new functional biomaterials (e.g., artificial muscle, biosensors) but also for understanding the principles of complex biological systems such as cartilage. Progress in the field requires an interdisciplinary effort to accomplish a better understanding of the structure and interactions of polyelectrolyte systems over multiple length and time scales.

## 6. Marine Microgels

Marine microgels are polymer networks formed by spontaneous assembly of biopolymers in the ocean with seawater entrapped in the swollen network [36]. Better understanding the behavior of these gels is particularly important because the ocean plays a critical role in global carbon cycling: it handles half of the global primary production of reduced organic carbon. As dissolved organic carbon is available for marine microorganisms (e.g., bacteria), the ocean is a huge repository of carbon [37,38,39]. Marine microgels exist in the ocean, i.e., an environment in which both mono- and multivalent counter-ions are present. The important role of counter-ions in the swelling of various polyelectrolyte gels was recognized a long time ago. Polymer physics provides valuable insights into the behavior of polyelectrolyte gels, whose knowledge is essential to understanding the role of marine biopolymers in carbon cycling. 

In the ocean, carboxylic acids are the most common negatively charged residues present in dissolved organic matter. Carboxylic acids contain a hydrogen ion that other ions (e.g., Na^+^, Ca^2+^, Mg^2+^) can replace. Counter-ions interact with oppositely charged sites. Polyanions with few available polyanionic sites are likely to form relatively unstable assemblies. An important feature of electrostatic interactions in polyelectrolytes is that the probability of forming links is proportional to the second power of the valence of the counter-ion [40]. For example, Fe^3+^ can induce self-assembly of the organic components in seawater at much lower concentrations and in shorter times than divalent counter-ions. The characteristic coagulating effect of low concentrations of Al^3+^ salts in seawater is another outcome that can be explained by the strong effect of high-valence polyions on the assemblies of dissolved carbon nutrients [41]. 

In this article, we use a didactic approach. First, we describe the fundamental properties of negatively charged polyelectrolyte gels. Then, experimental results are shown both for a synthetic (sodium polyacrylate, PA) and a biopolymer gel (DNA) swollen in mono- and multivalent salt solutions. The advantage of performing measurements on model polyelectrolyte hydrogels is that their structure is well defined (unlike the structure of most natural gels), which is essential for conducting a systematic and quantitative study. 

The following sections of this paper are organized as follows: After presenting the brief theoretical framework, describing the equilibrium swelling behavior of polyelectrolyte gels and the kinetics of gel swelling, the thermodynamic analysis of swelling equilibrium measurements conducted on gels swollen in solutions containing monovalent, divalent and trivalent salts is discussed. This is followed by the analysis of the swelling kinetics measurements. We compare the swelling kinetics of PA gels in salt-free water and in solutions containing both mono- and divalent cations. The results clearly indicate the important role of both ion exchange and ion diffusion in the development of complex structures consisting of coexisting shrunken and swollen regions. Finally, the main results are summarized in the conclusions.

We believe that understanding the behavior of solutions of charged biopolymers in near-physiological salt solutions will shed light on the mechanism of structure formation in various biological systems (e.g., organization of structural components of the cell and extracellular matrix). Although the experimental work presented here was conducted on model hydrogels, it is reasonable to assume that the response of these gels to changes in the ionic environment is similar to other polyelectrolyte gels, including marine microgels.

## 7. Theory

### 7.1. Swelling of Polyelectrolyte Gels

**Scheme 2:**
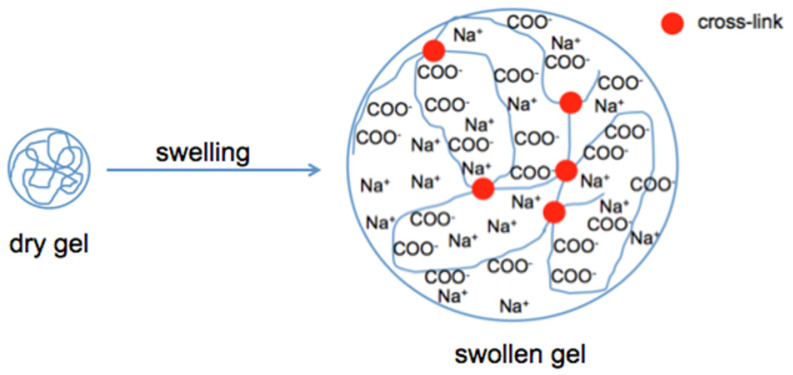
Swelling of a polyelectrolyte gel. Water uptake is driven by the osmotic pressure difference between the polymer network and the surrounding solution.

Polyelectrolyte gels are polymer networks in which the charged sites are fixed on the polymer chains, and the counter-ions in the surrounding liquid ensure electroneutrality. Due to the repulsive interaction between identically charged groups on the macromolecules, the dry network swells by absorbing solvent (e.g., water) molecules. The amount of water absorbed by a typical polyelectrolyte network can exceed 1000 times the weight of the dry polymer. However, the polymer network cannot be dissolved because of the presence of permanent cross-links. Changing the environment of the gel (salt concentration, pH, etc.) affects its swelling degree. As discussed earlier, with an increasing salt concentration, the counter-ions gradually screen the electrostatic repulsion between the charged groups on the polymer chains, and above a critical threshold, the salt concentration leads to the collapse of the gel. This effect can be reversed by removing the salt from the collapsed gel. The swelling process is illustrated in Scheme 2. 

The stability of a polyelectrolyte gel is the result of a delicate balance between several competing thermodynamic forces. At equilibrium, the free energy, ΔF_tot_, of the swollen network reaches a minimum. In the case of neutral polymer gels, ΔF_tot_ is the sum of the free energy of elastic deformation of the network chains, ΔFel, and the free energy of the mixing of polymer and solvent molecules, ΔFmix [4,13,42]. In the case of polyelectrolytes, however, there is an additional term, ΔFion, due to the presence of the counter-ions (Donnan contribution). Assuming that these terms are independent, we can write [4]

ΔF_tot_ = ΔF_el_ + ΔF_mix_ + ΔF_ion_ (1)

In an osmotic swelling experiment, the derivatives of the free energy components are measured, i.e.,

Π_tot_ = − ∂(ΔF_tot_/V_1_)/∂n_1_ = Π_el_ + Π_mix_ + Π_ion_ (2)

where Π_tot_ is the swelling pressure of the gel, Π_el_, Π_mix_ and Π_ion_ are the elastic, mixing and ionic contributions of Π_tot_, V_1_ is the molar volume of the solvent, and n_1_ is the number of moles of the solvent. 

For networks made of flexible polymer chains, the elastic contribution can be estimated from the theory of rubber elasticity [13,42]:

Π_el_ = −ARTν φ^1/3^ = −G (3)

where ν is the concentration of the elastic chains, φ is the volume fraction of the polymer, R is the gas constant, and T is the absolute temperature. The constant, A, depends on the functionality of the junctions and the topology of the network. Π_el_ can be expressed by the shear modulus, G, of the gel. 

The osmotic mixing pressure, Π_mix_, can be given by the Flory–Huggins expression:

Π_mix_ = −RT/V_1_ [ln(1−φ) + φ + χ_ _φ^2^ + χ_1_φ^3^] (4)

where χ_0_ and χ_1_ are constants (interaction parameters).

The ionic contribution is due to the difference in mobile ion concentrations inside and outside the polyelectrolyte gel [4], which gives rise to an osmotic pressure difference, Π_ion_, between the gel and the equilibrium solution. According to the theory of Donnan,

(5) $$\Pi_{\mathrm{ion}}=\mathrm{RT}\;\sum_{j=1}^{N}\;({\mathrm{cj}}^{\mathrm{gel}}\;-\;{\mathrm{cj}}^{\mathrm{sol}})$$

where cj_gel_ and cj^sol^ represent the concentrations of the ions in the gel and in the equilibrium solution, and N is the number of mobile ions in the system.

Recent experimental studies [43,44,45,46,47], as well as molecular dynamics simulations [48], indicated that in the presence of added salt, the Donnan contribution is very small, i.e., the first two terms of Equation (2) provide a satisfactory description of the osmotic properties of polyelectrolyte gels.

### 7.2. Kinetics of Gel Swelling

The kinetic theory of gel swelling is based on the concept of cooperative diffusion of the network polymer in a continuous medium [49,50]. According to the theory of Tanaka,

(6) $$u(r,\;t)/u(r,\;\infty )=\sum_{i}B_{i}\;\exp\;[-t/\tau_{i}]$$

where u(r, t) is the displacement vector, t is the swelling time, and τ_i_ is the relaxation time. The numerical factor B_i_ depends on the geometry of the gel and the ratio of the shear modulus G over the longitudinal osmotic modulus M_os_. This theory views gel swelling as a combination of pure diffusion and pure shear relaxation processes. The important role of the shear modulus is to keep the system in shape. Since during a shear relaxation process, there is no relative motion and, consequently, no friction between the polymer and the solvent, the gel can instantaneously adjust its shape, thereby minimizing nonisotropic deformation.

At a large t, the slowest term of Equation (6) dominates. For spherical gels, the theory predicts

d_t_ = d_∞_+ [d_0_−d_∞_] B_1_ exp(-t/τ_1_) (t > τ_1_) (7)

where d_t_ is the diameter of the gel at time t, d_0_ and d_∞_ are the initial and final (equilibrium) diameters, and τ_1_ is the relaxation time of the slowest mode in the swelling process.

Since gel swelling is a diffusion-controlled process, the rate of swelling (or shrinking) depends on the size of the sample. The collective diffusion coefficient D_c_ is given by

D_c_ = M_os_/*f* = *a*^2^/(β_1_^2^τ_1_) (8)

where *f* is the friction coefficient, *a* is the radius of the gel, and β_1_ is the function of G/M_os_. This collective mode of diffusion, which is distinct from the translational motion either of the individual solvent molecules or the polymer chains, governs the rate at which polymer and solvent molecules mutually exchange positions upon swelling or deswelling. Specifically, D_c_ defines the rate at which the solvent enters or leaves each elementary fluctuating volume. The size of this volume depends on the thermodynamic conditions, notably the polymer concentration and the salt content. Thus, the value of D_c_ reflects changes in the ionic environment. For neutral polymer gels, D_c_ is typically in the order of 10^−6^–10^−7^ cm^2^/s. 

## 8. Results and Discussion 

In this section, we show typical experimental findings for model polyelectrolyte gels. First, we focus on the effect of counter-ion valence on the macroscopic swelling behavior of sodium polyacrylate (PA) and DNA gels. We separate the elastic and mixing contributions of the swelling pressure and investigate the variation in these components with the ion valence and the ion concentration. 

### 8.1. Effect of Salts on the Swelling and Osmotic Behavior of Model Polyelectrolyte Gels

**Figure 1:**
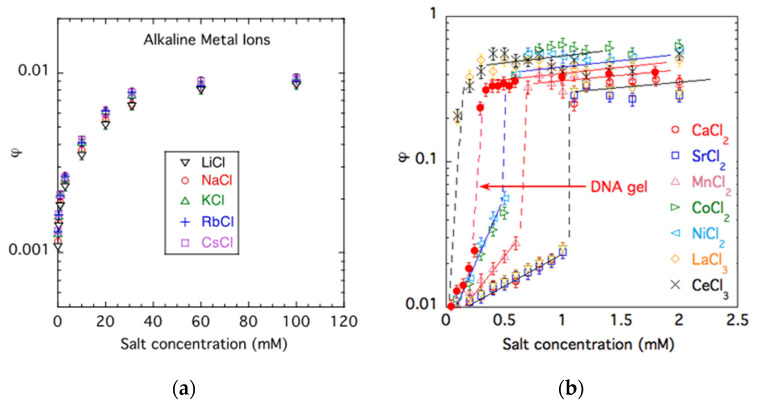
Effect of different monovalent (**a**) and multivalent salts (**b**) on the volume fraction φ of PA gels. Filled symbols show the dependence for a DNA gel. In (**b**), measurements were conducted in solutions containing 40 mM NaCl. Curves are a guide for the eye.

Figure 1 shows the dependence of the polymer volume fraction φ of PA hydrogels as a function of the salt concentration, c_salt_, in solutions of monovalent (Figure 1a) and multivalent salts (Figure 1b). Gel swelling is the greatest in salt-free solutions. The general trend is that the salt addition induces gel contraction because ions screen the repulsive electrostatic interactions between the charged groups on the polymer chains. However, the valence of counter-ions has a significant effect on the swelling behavior. In monovalent salt solutions (LiCl, NaCl, KCl, RbCl, CsCl), the polymer concentration increases from φ ≈ 0.001  to φ ≈ 0.01. It can also be seen that the effect of different divalent ions is similar, indicating that the ion valence, rather than the chemical nature of the cation, determines the swelling of the present anionic PA gels. 

In the presence of divalent counter-ions (Ca^2+^, Sr^2+^, Mn^2+^, Co^2+^, Ni^2+^) at a ‘critical concentration’ (or a critical ratio of divalent to monovalent cations), a reversible volume transition occurs. The sharp volume change with an increasing concentration of divalent counter-ions indicates that the transition is a highly cooperative process. Figure 1b also shows the data for a weakly cross-linked DNA gel swollen in CaCl_2_ solutions, which exhibits a qualitatively similar behavior to the PA gels, although the numerical values are different. In the PA gel, the volume transition occurs at approximately 1 mM CaCl_2_ concentration, while in the DNA gel, it takes place at a lower calcium ion content (≈0.25 mM CaCl_2_).

Trivalent cations shift the transition concentration toward lower salt concentrations. We note that the present trivalent counter-ions (Ce^3+^ and La^3+^) practically bind irreversibly to the polyanion. 

A volume change reflects the interplay between two main effects: attractive interactions among the polymer segments, which tend to shrink the gel, and repulsion of similarly charged species (either charged units of the polymer network or mobile ions). Consequently, any change in the mixing and elastic free energy contributions affects the polymer concentration. 

To reveal the effect of added salts on the equilibrium swelling of gels, the elastic and mixing contributions of the free energy should be separately investigated. First, we quantify the effect of ions on the elastic modulus of the gels. Then, we focus on the osmotic mixing component by conducting swelling pressure measurements on gels containing different salts. We compare the osmotic results for two entirely different gel systems: PA and DNA gels.

**Figure 2:**
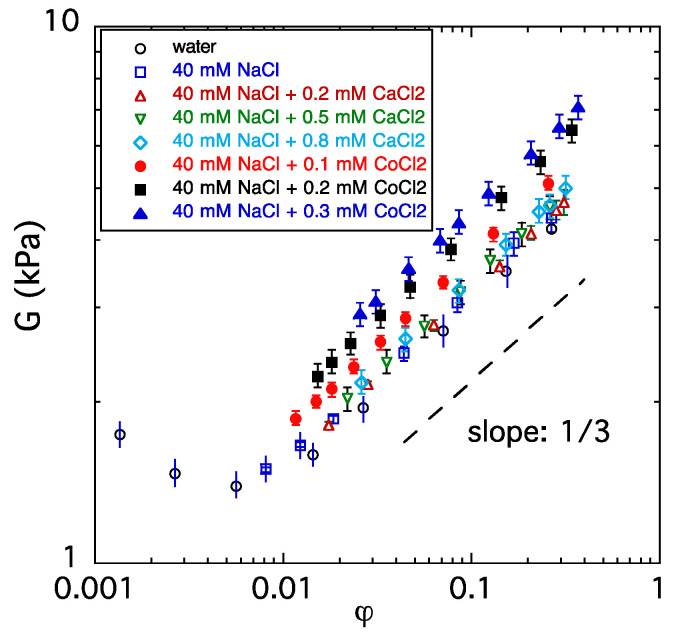
Variation in the shear modulus of PA gels swollen in different salt solutions and water.

It is often assumed that divalent cations form bridges between the charged groups on the polyelectrolyte network. The formation of ion bridges is expected to increase the apparent cross-link density and thus the elastic (shear) modulus of the gel. In Figure 2, the dependence of the shear modulus on the polymer volume fraction of PA gels measured in CaCl_2_ and CoCl_2_ solutions is shown. In CaCl_2_ solutions, all data points fall on a single curve, indicating that G is a function of the polymer concentration only, i.e., the concentration of Ca^2+^ ions does not modify the ‘effective’ cross-link density of the gel. At low and moderate volume fractions, G varies according to the power law prediction of the theory of rubber elasticity, i.e.,

G = G_o_φ^1/3^ (9)

where G_o_ is a constant (G_o_ = ARTν). Deviation from the theoretical dependence is observed only in the most swollen gels (without an added salt), where the shear modulus increases with the decreasing volume fraction. In such highly swollen gels, the finite extensibility of the network chains dominates, and the elastic response can no longer be described by the Gaussian elasticity theory. A similar upturn in the elastic modulus at high swelling degrees was observed previously in ionized acrylamide-sodium acrylate copolymer gels [51,52].

In general, alkaline earth metal ions cause gel contraction but do not form additional cross-links. Figure 2 also shows that when Ca^2+^ ions are replaced by Co^2+^ ions, the value of G increases with the increasing CoCl_2_ concentration (filled symbols). A probable explanation of the increase in the elastic modulus is complex formation between the Co^2+^ ions and the polyacrylate anion. 

**Figure 3:**
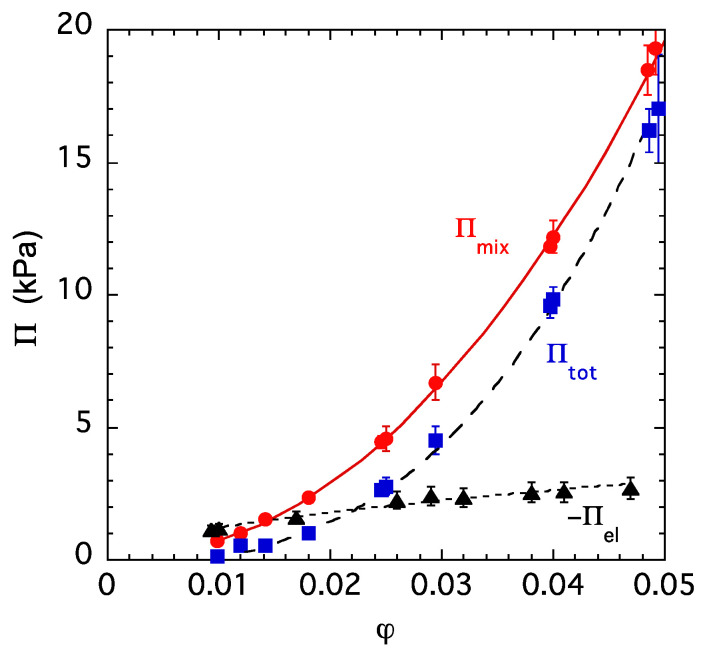
Variation in the swelling pressure Π_tot_ and its osmotic Π_osm_ and elastic Π_el_ components with the volume fraction of the polymer in a PA gel swollen in 40 mM NaCl solution.

Figure 3 shows the total swelling pressure of a PA gel, Π_tot_, and its elastic Π_el_ and mixing Π_mix_ components at a constant ion concentration (c_salt_: 40 mM NaCl solution). Π_tot_ was determined from osmotic stress measurements, Π_el_ was estimated from the shear modulus (Π_el_ = −G) and Π_mix_ was calculated using the relationship Π_mix_ = Π_tot_ + G. Both Π_tot_ and Π_mix_ increase while Π_el_ decreases with the increasing polymer volume fraction. The continuous curve is the least squares fit of Equation (4) to the Π_mix_ data, which yields, for the interaction parameters, χ_0_ = 0.448 ± 0.001 and χ_1_ = 0.21 ± 0.01.

**Figure 4:**
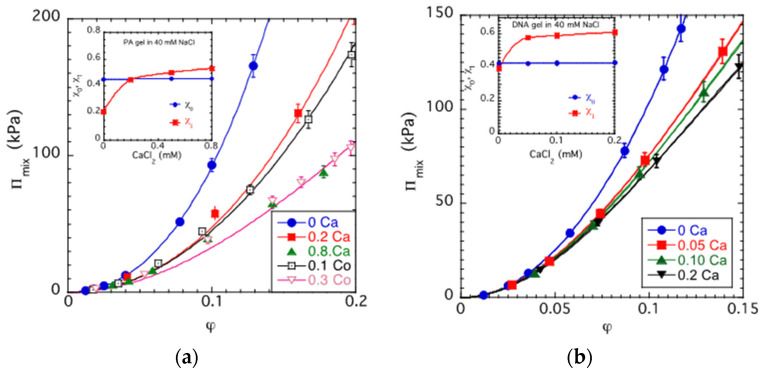
Mixing pressure, Π_mix_, vs. polymer volume fraction, φ plots for PA (**a**) and DNA gels (**b**) in different salt solutions. The continuous lines show the fits of Equation (4) to the experimental data. Insets: variation in χ and χ_1_ as a function of the CaCl_2_ concentration in PA and DNA gels swollen in 40 mM NaCl.

It is reasonable to assume that specific interactions between the counter-ions and the carboxylate groups may modify the mixing contribution of the network free energy. The dependence of Π_mix_ as a function of φ is shown in Figure 4a for PA gels swollen in 40 mM NaCl solutions containing different amounts of CaCl_2_ or CoCl_2_. It can be seen that both the chemical type and the concentration of the cations affect the mixing pressure. Π_mix_ (i) decreases with the increasing concentration of divalent cations and (ii) depends on the chemical type of the cation; the effect of Co^2+^ ions is qualitatively similar but significantly greater than that of the Ca^2+^ ions. The lines through the experimental data points are least squares fits to Equation (4). As shown in the inset, the addition of divalent counter-ions causes a very weak increase in the value of χ over the entire concentration range explored, while χ_1_ strongly increases as the divalent ion concentration increases and thereafter exhibits a slow increase. Figure 4b shows similar data for a DNA gel swollen in 40 mM NaCl solution with different CaCl_2_ contents. Again, Π_mix_ decreases with increasing Ca^2+^ concentration. The variation in χ_ _ and χ_1_ with the CaCl_2_ concentration is similar in the DNA and PA gels, indicating that gel swelling is primarily governed by the electrostatic interactions in the system, and the role of the chemistry of the polymer chains is less important. 

In summary, based on the effect of ions on the apparent cross-link density and the osmotic mixing contribution, a hierarchy can be established in the interaction strength according to the chemical group to which the ions belong. Alkali metal ions and alkaline earth metal ions do not considerably affect the elastic properties of PA hydrogels. The effects of alkaline earth metal salts can be attributed to modification of the mixing free energy. Experimental data indicate that the addition of CaCl_2_ has little effect on χ but significantly increases χ_1_. Transition metal ions (Co^2+^, Ni^2+^) form complexes with the polyanion, which can be interpreted as additional cross-links, as indicated by the increase in the elastic modulus. In these gels, cations affect both the elastic and mixing free energy terms. Trivalent cations (La^3+^ and Ce^3+^) practically bind irreversibly to the polyanion. 

Gel swelling is primarily governed by the effect of ions on the electrostatic interactions among the charged groups of the polymer network. With the increasing concentration of the added salt, the repulsive interaction is gradually screened, and above a critical concentration of the counter-ions, a sudden volume change takes place. The decisive factor in the volume transition is the ion valence, while the chemical type of the ions and the chemical composition of the polymer network are of secondary importance. 

### 8.2. Kinetics of Polyelectrolyte Gel Swelling in Salt Solutions

**Figure 5:**
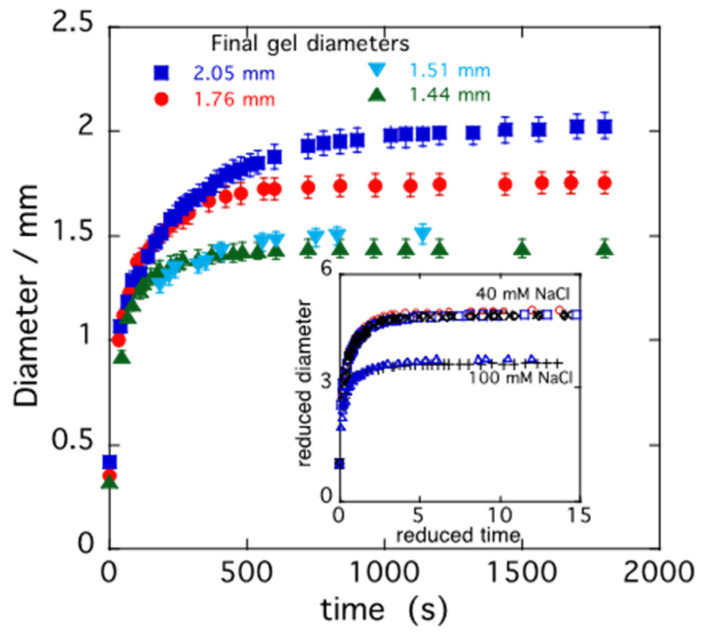
Swelling kinetics data of PA gels in 40 mM NaCl solution. Inset: reduced gel diameter d/d_0_ vs. reduced time t/τ_1_ for PA gels swollen in 40 mM NaCl and 100 mM NaCl solutions.

In Figure 5, the swelling kinetics data measured in 40 mM NaCl solution for spherical PA gels of different sizes are shown [53]. All samples exhibit a qualitatively similar behavior: the gel size monotonically increases as a function of time and asymptotically approaches a plateau. As expected, smaller gels swell faster. The inset in Figure 5 illustrates the variation in the reduced gel diameter d_t_/d_o_ with the reduced time t/τ_1_ for the same gels, as in the main figure (upper curves), and for gels swollen in 100 mM NaCl solutions (lower curves). At each salt concentration, the data points fall on a master curve. The height of the plateau region, corresponding to the equilibrium concentration of the fully swollen gels, decreases with the increasing salt concentration.

The analysis of the swelling kinetics data was performed using the linearized form of Equation (7):

Y = ln [(d_∞_ − d_t_)/(d_∞_ − d_0_)] = ln B_1_ − t/τ_1_ (10)

From the intercept of the long-time linear extrapolation of the logarithmic plot, and from the slope of the straight line, B_1_ and τ_1_, respectively, can be determined. Once B_1_ is known, β can be found since both B_1_ and β depend on G/M_os_, and this dependence has been established numerically for spheres in the literature [50]. 

**Figure 6:**
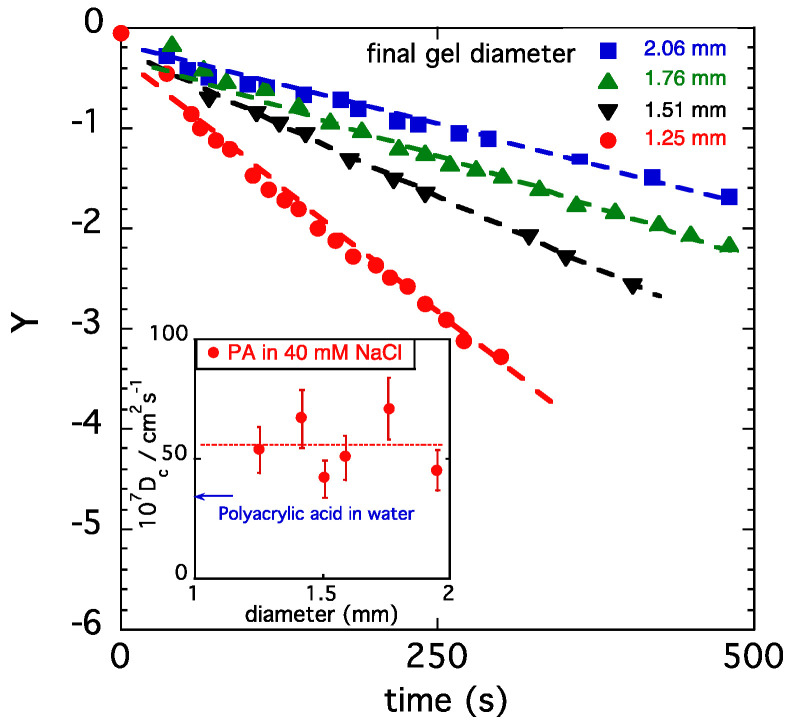
Variation in Y in Equation (10) as a function of the swelling time for PA gels swollen in 40 mM NaCl solution. Dashed curves are least squares fits to Equation (10). Inset: collective diffusion coefficient D_c_ of different hydrogels.

In Figure 6, the quantity Y is plotted as a function of time for spherical gels with different initial diameters. At higher values of t, all gels exhibit a linear behavior, as predicted by Equation (10), in agreement with the expectation that the relaxation time decreases with decreasing gel size. B_1_ is, however, the same for the four gel samples shown in Figure 5, indicating that the ratio of the moduli G/M_os_ is independent of the gel size. The values of τ_1_ and B_1_ obtained from the fits were used to calculate D_c_.

In the inset, the cooperative diffusion coefficient D_c_ is presented for PA gels of different sizes. In Figure 6, the value of D_c_ reported for a polyacrylic acid gel [51] measured in salt-free water by dynamic light scattering is also displayed. The present result shows that D_c_ in 40 mM NaCl solution exceeds the value measured in pure water by approximately 30%. 

**Figure 7:**
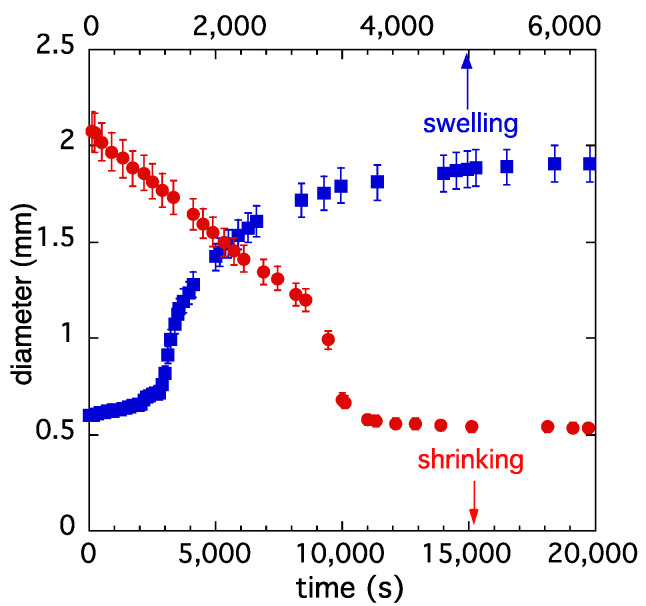
Kinetics of swelling (squares) and shrinking (circles) of a PA hydrogel. Gel was shrunken in 40 mM NaCl solution containing 10 mM CaCl_2_ solution and reswollen in 40 mM NaCl solution (without CaCl_2_).

The presence of divalent cations in the solution affects the swelling/shrinking kinetics of PA hydrogels. Figure 7 shows the variation in the diameter of a PA gel after the addition of 10 mM CaCl_2_ to the surrounding 40 mM NaCl solution. In the shrinking process, three different stages can be clearly distinguished. In the first stage, the swelling degree decreases slowly, practically linearly with the time. In the course of this process, the Ca^2+^ concentration progressively increases from the surface of the gel to the center. At a certain Ca^2+^ concentration, a volume transition occurs. First a collapsed layer is formed on the surface, which expands as the Ca^2+^ front moves towards the center. In this stage, the collapsed network coexists with the swollen gel. 

Figure 7 also shows the reswelling of the collapsed PA gel in free Ca^2+^ 40 mM NaCl solution. First, the Ca^2+^ ions diffuse from the collapsed gel into the surrounding NaCl solution, producing a moderate increase in gel swelling. When the Ca^2+^ content of the gel falls below the transition concentration, a rapid increase in the swelling degree takes place, followed by a plateau region, which slightly increases as Ca^2+^ ions gradually leave the gel.

**Figure 8:**
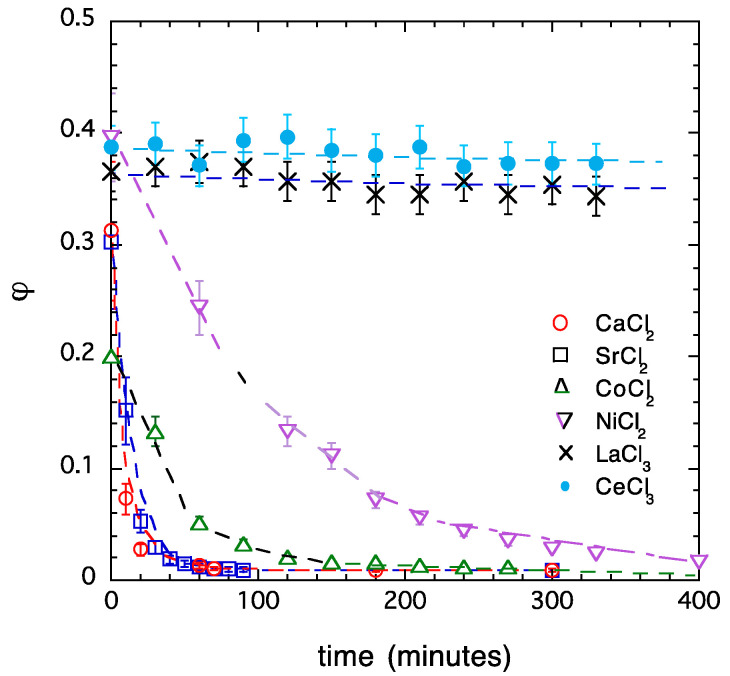
Kinetics of reswelling of PA gels deswollen in multivalent salt solutions. The gels were immersed in 40 mM NaCl solution.

Measurement of the swelling rate makes it possible to estimate the relative ‘stability’ of the collapsed state of the gels. Figure 8 compares the rate of water uptake of PA gels containing various multivalent cations. To ensure a fair comparison between the swelling rates, prior to the swelling experiment, identical gel samples were collapsed in salt solutions containing 2 mM multivalent salt in 40 mM NaCl. Then, the collapsed gels were transferred into 40 mM NaCl solution. The reswelling of gels collapsed in solutions of alkaline earth metal salts (CaCl_2_, SrCl_2_) was the fastest, followed by gels deswollen in solutions of transition metal salts (CoCl_2_, NiCl_2_). For gels with trivalent cations (La^3+^, Ce^3+^), no appreciable reswelling was observed, even after 3–4 weeks. On the basis of the swelling curves shown in Figure 8, the stability of the collapsed state varies in the order La^3+^ ≈ Ce^3+^ > Ni^2+^ > Co^2+^ > Ca^2+^ ≈ Sr^2+^. These results are in qualitative agreement with the results of the osmotic and mechanical observations discussed above.

## 9. Conclusions

The effects of different cations on the osmotic behavior and swelling kinetics of chemically cross-linked PA and DNA gels were discussed. The addition of multivalent cations to polyelectrolyte gels swollen in NaCl solution led to a volume transition in these gels in a biologically relevant concentration range. The electrostatic repulsion between the charged network chains decreased because multivalent ions more efficiently compensate the charge on the polyanion than monovalent counter-ions. 

A hierarchy was established in the interaction strength between cations and polyelectrolyte chains according to the chemical group to which the cation belongs. Alkali metal ions (Li^+^, Na^+^, K^+^, Rb^+^, Cs^+^) practically moved freely all over the entire network. Alkaline earth metal ions (Ca^2+^, Sr^2+^) promoted weak associations among the network chains, while transition metal ions (Co^2+^, Ni^2+^) formed stronger (but not irreversible) bridges. Trivalent cations (La^3+^ and Ce^3+^) practically bound irreversibly to the polyanion. 

Analysis of the osmotic results on the basis of the Flory–Huggins formalism provided an empirical description of the effect of Ca^2+^ ions on the osmotic pressure both below and in the vicinity of the volume transition. Ca^2+^ ions primarily affected the third-order interaction parameter, which strongly increased with the increasing Ca^2+^ concentration in the surrounding solution, while the second-order interaction parameter only weakly varied. The gradual increase in the interaction parameters with the increasing CaCl_2_ concentration created the condition for a volume transition. The reversible nature of the volume transition and the absence of a measurable effect of Ca^2+^ ions on the elastic modulus indicated that calcium ion binding is not permanent.

It was shown that the kinetics of the swelling of PA gels in NaCl solution was characterized by a collective diffusion coefficient, which is independent of the initial size of the gel particles. When a gel swollen in NaCl solution is immersed in a solution that contains Ca^2+^ ions, three different stages in the shrinking process can be distinguished. First, the swelling degree slowly decreases with the time. This is followed by a sudden shrinking due to the volume transition. Then, gel contraction continues until the fully collapsed state is reached. When the gel is reswelling from the collapsed state in NaCl solution, first, the swelling degree slowly increases. This is followed by a steep increase corresponding to the volume transition. The last stage is a slow swelling, while the Ca^2+^ concentration gradually decreases in the gel.

In this article, we demonstrated that an ion-induced volume transition in polyelectrolyte gels exhibits a universal behavior, which is practically independent of the molecular details. Although the molecular mechanism responsible for the volume transition is not fully understood, the present results clearly indicate the important role of ion exchange and ion diffusion in the development of complex structures consisting of coexisting shrunken and swollen regions. Understanding the organization of charged macromolecules in near-physiological salt solutions may shed light on the mechanism of structure formation in biological systems, e.g., organization of nucleic acids or other structural elements of the cell or the extracellular matrix. The complexity of the structure and interactions in living systems makes it difficult to perform conclusive experiments under well-controlled conditions. Systematic studies conducted on model gel systems can provide vital insight into the nature of certain universal phenomena that play a role in biological systems. This understanding cannot be obtained from measurements conducted on biological samples because their composition and physical properties cannot be independently and systematically varied as they can be in model systems. 

## 10. Materials and Methods

### 10.1. Gel Preparation

Sodium polyacrylate gels were produced by free radical copolymerization of partially neutralized acrylic acid and N,N′-methylenebis(acrylamide) cross-linker in aqueous solution according to a procedure described previously [44]. The monomer concentration was 30% (*w/w*), and 35% of the monomers were neutralized by sodium hydroxide before polymerization. Dissolved oxygen was removed by bubbling nitrogen through the solution. Then, ammonium persulfate (0.5 g/L) was added to initiate the polymerization reaction. Gelation was carried out at 80 °C.

Gel beads were produced by polymerization in silicone oil (viscosity: 1000 cPs) that was previously degassed with nitrogen. Spherical droplets (diameter < 1 mm) of the mixture were injected into the silicone oil. After gelation, gel samples were completely neutralized and washed in deionized water to remove all extractable materials (e.g., sol fraction). Water was renewed every day for two weeks.

For the mechanical measurements, cylindrical gel specimens (1 cm height, 1 cm diameter) were produced in a special mold using the same cross-linking procedure. Gel cylinders were neutralized and washed for several weeks with deionized water before the experiments.

DNA gels were produced from deoxyribonucleic acid sodium salt (Na-DNA from salmon testes, Sigma-Aldrich, St. Louis, MO, USA). According to the manufacturer, the % G-C content of this DNA was 41.2%, and the melting temperature was reported to be 87.5 °C in 0.15 M sodium chloride plus 0.015 M sodium citrate. The molecular weight determined by ultracentrifugation was 1.3 × 10^6^, corresponding to approximately 2000 base pairs. First, DNA was dissolved in a HEPES buffer (pH = 7.0); then, the solutions were dialyzed against distilled water. DNA gels were produced by cross-linking [45] with ethylene glycol diglycidyl ether (2%) at pH = 9.0 using TEMED to adjust the pH. The DNA concentration at cross-linking was 3% (*w/w*). The gels were equilibrated in NaCl solutions containing different amounts of CaCl_2_ (0–0.2 mM).

### 10.2. Osmotic Stress Measurements

Osmotic stress measurements were conducted on PA and DNA gels by aqueous solutions of poly(vinyl pyrrolidone) (PVP, molecular weight: 29 kDa). The osmotic pressure of the PVP solution was known from independent measurements [54,55]. The swollen network was separated from the solution by a semipermeable membrane, which prevented the penetration of the polymer molecules into the gel. At equilibrium, the swelling pressure of the gel inside the dialysis bag is equal to the osmotic pressure of the PVP solution outside. The size and the weight of the gel samples were measured when equilibrium was attained. The reversibility of the deswelling process was checked by transferring the deswollen gels into PVP solutions of different concentrations. No significant difference was found between swelling degrees obtained by decreasing or increasing the osmotic pressure of the equilibrium solution. 

When gel beads were equilibrated with salt solutions, it was assumed that the salt concentration in the liquid phase surrounding the gel sample was unchanged (infinite bath).

### 10.3. Swelling Kinetics Measurements

Gel beads prepared according to the procedure described above were placed into a Petri dish containing salt solution. The diameter of the gel was measured as a function of time under a Leica MZ 12 stereomicroscope using a calibrated scale. Swelling kinetics measurements were carried out in NaCl solutions containing different amounts of CaCl_2_ at room temperature [53].

### 10.4. Elastic Modulus Measurements

Uniaxial compression measurements were performed on gel cylinders in equilibrium with salt solutions using a TA.XT2I HR Texture Analyser (Stable Micro Systems, Vienna Court, UK). This apparatus measures the deformation (±0.001 mm) as a function of the applied force (±0.01 N). Measurements were performed at deformation ratios of 0.7 < Λ < 1. Typical sample sizes were: height 0.5 to 2 cm, diameter 0.5 to 2 cm. The elastic (shear) modulus, G, was calculated from the nominal stress, σ (force per unit undeformed cross-section), using the equation [43]

σ = G (Λ−Λ^−2^) (11)

where the deformation ratio is Λ = L/L_o_ (L and L_o_ denote the heights of the deformed and undeformed gel cylinders, respectively).

Both swelling and mechanical measurements were carried out at 25 ± 0.1 °C.

## Data Availability

The data that support the findings of this study are available from the corresponding author upon reasonable request.
